# Video-capillaroscopy, a promising tool for the clinical evaluation of patients with chronic venous insufficiency

**DOI:** 10.3389/fmed.2023.1144102

**Published:** 2023-02-16

**Authors:** Patrick H. Carpentier, Corine Trolliet, André Cornu-Thénard, Rémy Chamberod, Jérôme Laurès, Janick Noilhetas, Eric Chauvin

**Affiliations:** Centre de Recherche Universitaire de La Léchère, Université Grenoble Alpes, Aigueblanche, France

**Keywords:** venous insufficiency, capillaroscopy, microcirculation, skin, microangiopathy, post-thrombotic syndrome

## Abstract

**Background:**

The cutaneous microangiopathy plays a key role in the development of the skin clinical lesions of venous insufficiency. Capillaroscopy allows a non-invasive observation of the superficial skin capillaries of the lower leg, which have previously been shown to be altered in patients with advanced venous disease. As it is now available in a friendly, easy to handle way through modern video devices, we report our findings in a short series of patients with C3–C5 chronic venous disorders using this technique.

**Methods:**

A total of 21 patients with venous insufficiency (C3–C5 on at least one leg) underwent a capillaroscopic examination of both legs and pictures recorded from the sites of the most severe venous skin lesions. This was performed with a CapXview handheld video-capillaroscope (×100 magnification), allowing easy manual measurement of maximum capillary bulk diameter and capillary density.

**Results:**

Dramatic changes in capillary density, size, and shape were easily observed at the site of the venous skin lesions. A significant negative linear relationship was found between capillary density and the “C” classes (*r* = −0.45; *P* < 0.001). A significant negative correlation was also found between capillary density and bulk diameter (*r* = −0.52; *P* < 0.001). The area under the ROC curve for the mathematical prediction of venous skin changes by capillary density was 0.842, which shows the strength of the link between the microvascular and the clinical status.

**Conclusion:**

Video-capillaroscopy allows a direct observation of the cutaneous venous microangiopathy and provides the possibility to measure capillary density which allows its quantification. This simple to use technique shows the potential for a more precise follow-up and treatment evaluation of the cutaneous consequences of venous disease, which remains to be further investigated.

## Introduction

Chronic venous disease of the lower limbs is one of the most prevalent pathological conditions in industrialized countries. The severity of its consequences is related to the damage in the cutaneous tissue that can eventually lead to leg ulcers, which induces a huge burden for the health care systems (1, 2). In patients with this condition, the cutaneous microangiopathy plays a key role in the development of the skin clinical lesions (3).

Capillaroscopy allows a non-invasive observation of the superficial skin capillaries of the lower leg, which have previously been shown to be altered in patients with advanced venous disease (4–9). As it is now available in a friendly, easy to handle way through modern video devices (10, 11), we report our findings in a short series of patients with C3–C5 chronic venous disorders using this technique where we tried to evaluate if this examination was feasible in a regular outpatient setting and whether it is able to quantify the venous microangiopathy.

## Materials and methods

Patients with venous insufficiency (CEAP “C” classes C3–C5) underwent a capillaroscopic examination as part of the physical examination of their lower legs during a balneotherapeutic treatment course in the spa resort of La Léchère (Savoie – France), with a primary aim to more carefully evaluate the skin changes related to chronic venous disease. Twenty-one consecutive patients who were graded at least C3 in one lower limb gave a written consent for the use of their data and clinical pictures for clinical research.

**FIGURE 1:**
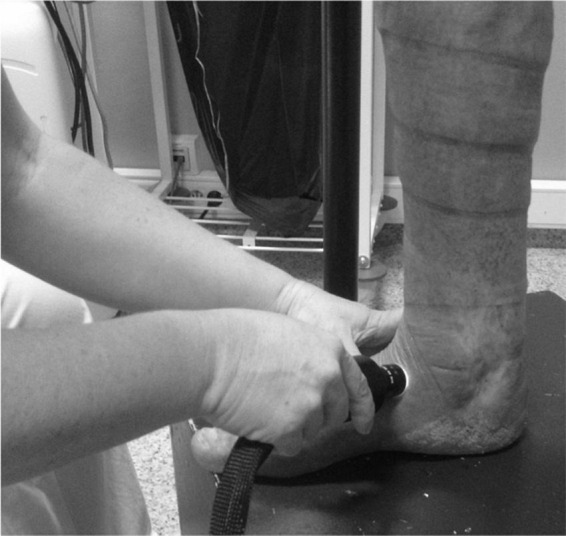
Capillaroscopic examination of the lower leg with the CapXview handheld video-capillaroscope.

This capillaroscopy examination was performed on the patient in standing position “Figure 1” on both legs, and pictures recorded at the site of the most severe venous skin lesion of the leg or at the medial malleolus level if no significant skin lesion was detected. We used a CapXview handheld video-capillaroscope, with ×100 magnification objective, which provided 3 × 2.2 mm pictures.

**FIGURE 2:**
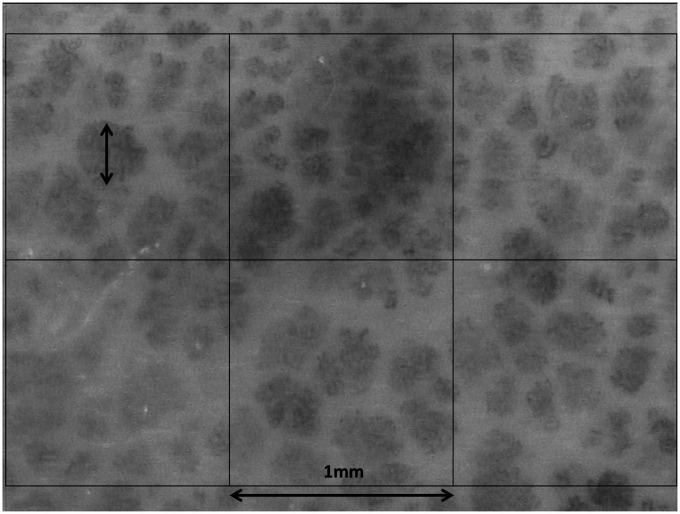
Capillaroscopic picture obtained with the CapXview handheld video-capillaroscope at ×100 magnification, with superimposed 1 × 1 mm allowing manual measurement of capillary density and of the maximum capillary bulk diameter.

Measurement of capillary density/mm^2^ and maximum loop diameter was performed by a single blinded investigator (PHC) on de-identified pictures as follows “Figure 2”: six areas, each of 1 mm^2^, were automatically superimposed to the capillaroscopy image. The number of capillaries in each area was counted manually; when a capillary was crossing the limit between two or three areas, it was counted in the area where stayed its barycenter. The mean value of the six measurements was taken into account. The maximum diameter of the largest capillary bulk in the area was also measured.

Statistics were performed with IBM SPSS Statistics version 27, using analysis of variance, paired Wilcoxon tests and ROC analysis software. A *P* < 0.05 alpha risk threshold level was considered significant. Descriptive statistics are given as mean (SD).

## Results

Data were obtained from eleven females and ten men, aged 71 + 7 years. Thirteen of them had a previous history of deep vein thrombosis. Fourteen legs showed no venous insufficiency (C0–C2), nine were C3, ten C4a, five C4b and four C5 (as the examination took place before the publication of the 2020 CEAP revision, corona phlebectatica – C4c – was not taken into account). Regarding ultrasound duplex evaluation, 12 limbs showed deep venous reflux, 27 had saphenous reflux, and no significant reflux was found in thirteen limbs. The capillaroscopic examination was easily performed in all patients, with an ergonomics of the procedure very close to a Duplex examination.

**FIGURE 3:**
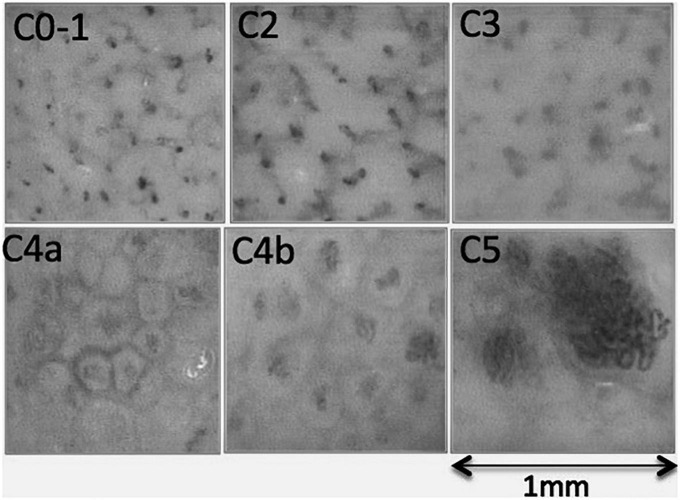
Capillaroscopic pictures illustrating the typical findings of each CEAP “C” class. C0, normal skin capillaroscopy with a “dots and comas” pattern, also found in most C1 and C2 limbs; C3, blurred picture related to the presence of edema; C4a, increased size of dermal papillae underlined by pigmentation, associated with increased tortuosity of capillaries leading to increased diameter of capillary bulk, and decreased capillary density; C4b, further increase of capillary tortuosity and bulk diameter with a marked reduction of capillary density; C5, extreme reduction of capillary density and huge capillary bulks sometimes with glomerulus-like shape.

**FIGURE 4:**
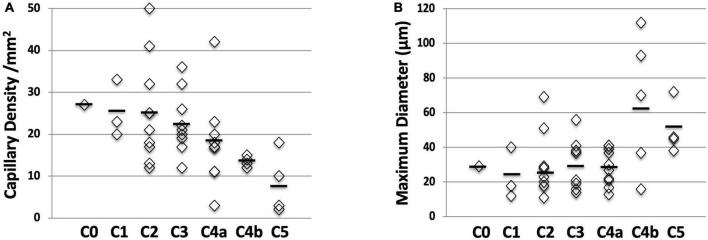
Relation between capillary density and diameter with CEAP “C” classes. **(A)** Capillary density shows a linear increase with increasing CEAP “C” classes (*P* < 0.001). **(B)** Capillary bulk diameter is increasing with increasing CEAP “C” classes but this does not reach statistical significance (*P* = 0.16).

**FIGURE 5:**
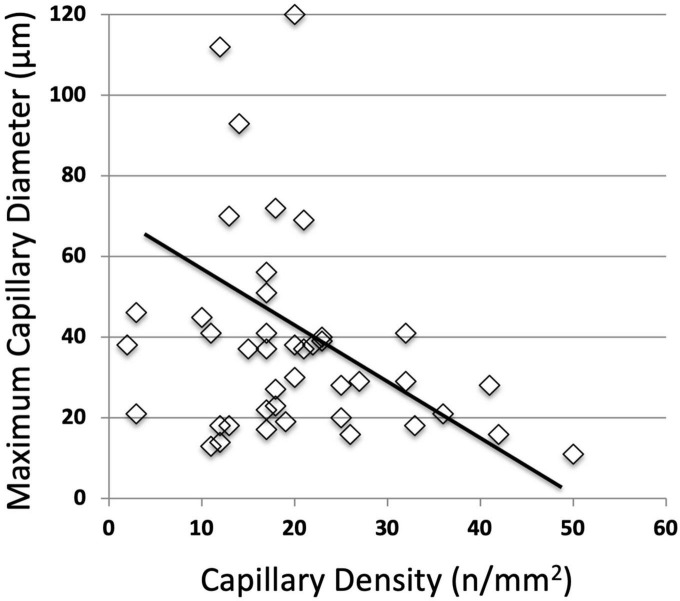
Negative correlation between capillary density and capillary bulk diameter (*r* = –0.52; *P* < 0.001).

**FIGURE 6:**
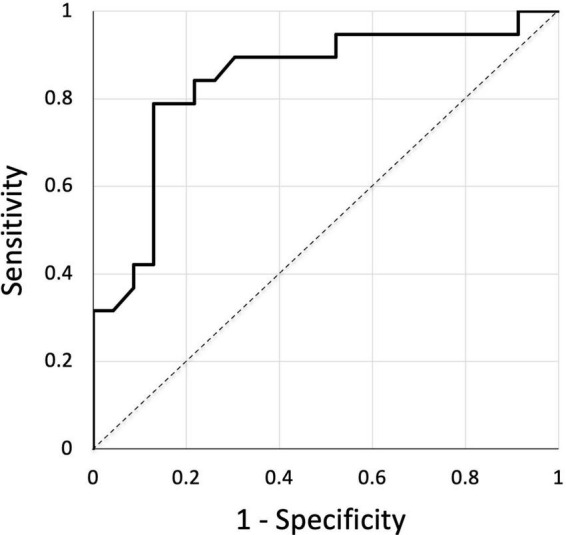
ROC curve showing the mathematical prediction of venous skin changes (C4–C5) for the different levels of capillary density. Area under the curve is 0.842.

Dramatic changes in capillary density, size and shape were observed at the site of the venous skin lesions as illustrated in “Figure 3.” A significant negative linear relationship between capillary density and the “C” classes (*r* = −0.45; *P* < 0.001) is shown on “Figure 4A.” A significant negative correlation was also found between density and diameter (*r* = −0.52; *P* < 0.001) as shown in “Figure 5.” However, the relationship with capillary bulk diameter with “C” classes was not found significant (“Figure 4B”). The ROC curve for the mathematical prediction of venous skin changes (C4–C5) by capillary density is shown on “Figure 6”; the C parameter (area under the curve) was as high as 0.842.

## Discussion

Our most interesting finding is that the capillaroscopy of the lower leg is indeed easily performed with the handheld video-capillaroscope, and able to provide pictures of the nutritional capillaries of the skin and to clearly show the venous microangiopathy in those patients with clinical signs of venous insufficiency. This microangiopathy is basically associating a decrease in capillary density and an increased diameter of the capillary bulk, and can be easily quantified. In this work, quantification was achieved through manual measurements of capillary density, which allows to avoid any detection artifact, but is time consuming. The use of digital image analysis systems would certainly easily improve the practicality of the quantification process.

Our quantitative results are interesting since they show a strong relation between the capillary density and the clinical severity of the clinical cutaneous consequences of venous insufficiency. The number of limbs examined is small, and we do not have a control group with skin lesions of different origin in order to evaluate the specificity of these microangiopathic patterns. In this respect, the ROC curve does not show the diagnostic reliability of the techniques, but we think it clearly demonstrates the strength of the quantitative relation between the decrease in capillary density and the clinical severity of the venous disease as evaluated with the world wide accepted CEAP “C” instrument. In previous work using classic capillaroscopy through vital microscopy, Franzeck et al. (5) were able to show the relationship between capillary density and TcPO_2_ in the lower leg of patients with chronic venous insufficiency. Their findings, as well as the inverse correlation between capillary density and diameter of capillary bulk we found, also suggest that the increases in capillary tortuosity and convolutions measured through the increased diameter of capillary bulk are probably related to hypoxia induced neo-angiogenesis.

The association of a decrease in capillary density and the risk of skin ulceration is already well documented in patients with systemic sclerosis, where nailfold capillaroscopy was able to show a similar relationship at the nailfold, the place where capillaroscopy is most often used in clinical practice (12, 13), and that refers to the well-known principle of August Krogh that the most crucial parameter for the oxygen delivery to the tissue is the oxygen diffusion distance to the closest functioning capillary (14).

## Conclusion

In conclusion, the easy-to-handle video-capillaroscopes allows us to evaluate the skin microcirculation in the lower leg where it is damaged in patients with severe chronic venous disease. This opens the way for a potential routine evaluation of this important pathogenetic mechanism with important applications for the early detection of venous disease decompensation, as well as for a more precise follow-up and treatment evaluation of the patients with high risk of leg ulcers. Further works exploring the potential and limits of this technique are strongly needed.

## Data availability statement

The raw data supporting the conclusions of this article will be made available by the authors, without undue reservation.

## Ethics statement

Ethical review and approval was not required for this retrospective study on human participants in accordance with the local legislation and institutional requirements. The patients/participants provided their written informed consent for the use of their data and clinical pictures.

## Author contributions

PC: study design, statistical analysis, and manuscript writing. PC and CT: study organization and manuscript editing. PC, CT, RC, AC-T, JL, JN, and EC: data gathering. CT: data management. All authors contributed to the article and approved the submitted version.
